# Retraction: The Consequences of COVID-19 on Breast Cancer Screenings in an Underserved Urban Population and the Screening Access of Value for Essex Program’s Efforts to Control the Damage

**DOI:** 10.7759/cureus.r77

**Published:** 2023-09-08

**Authors:** William J Lee, Yash Shah, Nidhi Patel, Albert Ku, Anibian Rodriguez, Magdalena Salvador

**Affiliations:** 1 Radiology, Rutgers University New Jersey Medical School, Newark, USA; 2 Radiology, Drexel University College of Medicine, Philadelphia, USA

This article has been retracted at the request of the authors. All authors consent to this retraction and together they have provided the following explanation:

The members of the SAVE Program who were responsible for collecting and distributing the data used for Table 1 were not properly accredited. This also includes those involved in the execution of the program and the calling project. It is imperative that we maintain our integrity as members of the medical community, and allowing the article to remain accessible without properly crediting those essential to its creation would be an utmost violation of our values.

